# Pericentromeric location of the telomeric DNA sequences on the European grayling chromosomes

**DOI:** 10.1007/s10709-013-9740-7

**Published:** 2013-10-11

**Authors:** K. Ocalewicz, G. Furgala-Selezniow, M. Szmyt, R. Lisboa, M. Kucinski, A. M. Lejk, M. Jankun

**Affiliations:** 1 Department of Ichthyology, University of Warmia and Mazury in Olsztyn, ul. Oczapowskiego 5, 10-718 Olsztyn, Poland; 2 Department of Lake and River Fisheries, University of Warmia and Mazury in Olsztyn, Olsztyn, Poland; 3 Department of Fish Biology and Pisciculture, University of Warmia and Mazury in Olsztyn, Olsztyn, Poland; 4 Faculty of Science and Technology, University of Algarve, Faro, Portugal

**Keywords:** *Thymallus*, Salmonidae, Fish cytogenetics, Heterochromatin and chromosome polymorphism, Interstitial telomeric sites, FISH

## Abstract

The chromosomal characteristics, locations and variations of the C-band positive heterochromatin and telomeric DNA sequences were studied in the European grayling karyotype (*Thymallus thymallus*, Salmonidae) using conventional C-banding, endonucleases digestion banding, silver nitrate (AgNO_3_), chromomycin A_3_ and 4′,6-diamidino-2-phenylindole staining techniques as well as fluorescence in situ hybridization (FISH) and primed in situ labelling. Original data on the chromosomal distribution of segments resistant to *Alu*I restriction endonuclease and identification of the C-banded heterochromatin presented here have been used to characterize the grayling karyotype polymorphism. Structural and length polymorphism of the chromosome 21 showing a conspicuous heterochromatin block adjacent to the centromere seems to be the result of the deletion and inversion. Two pairs of nuclear organizer regions (NOR)-bearing chromosomes were found to be polymorphic in size and displaying several distinct forms. FISH with telomeric peptide nucleic acid probe enabled recognition of the conservative telomeric DNA sequences. The karyotype of the thymallid fish is thought to experienced numerous pericentric inversions and internal telomeric sites (ITSs) observed at the pericentromeric regions of the six European grayling metacentric chromosomes are likely relics of the these rearrangements. None of the ITS sites matched either chromosome 21 or NOR bearing chromosomes.

## Introduction

The common ancestor of the Salmonidae family experienced a whole genome duplication (WGD) event between 25 and 100 million years ago (Allendorf and Thorgaard 1984). The hypothetical karyotype of the first tetraploid ancestor has been proposed to comprise 96 uni-armed chromosomes (Phillips and Ráb 2001). To recover disomic segregation, genomes of the extant salmonid fishes have undergone rediploidization process including numerous chromosomal rearrangements and leading to formation of many different karyotypes with chromosome number ranging from 52 to 102 (NF = 72–168) (Phillips and Ráb 2001). In the contrary to the species from Salmoninae and Coregoninae subfamilies, graylings (Thymallinae) are rather poorly cytogenetically studied salmonids and only three species have been examined to date: *Thymallus thymallus* (Nygren et al. 1971; Kalat et al. 1988; Jankun et al. 2003), *T. arcticus* (Makoedov 1982; Severin 1986) and *T. grubii* (Makoedov 1987). Characteristics of the grayling karyotype differ from what is observed in the other salmonids. While most of the salmonids show chromosome numbers that are reduced when compared to the chromosome number in the their hypothetical tetraploid ancestor, numbers of the chromosomes and the chromosome arms in graylings range from 98 to 102 and from 146 to 168, respectively (Arai 2011). Moreover, grayling metacentric and sub-metacentric chromosomes that are in the prevalence in the Thymallinae karyotypes are much smaller than bi-armed chromosomes described in other salmonids. Increase of the chromosome arm number and retain of the chromosome number close to the karyotype of the hypothetical tetraploid ancestor of the Salmonidae suggest thymallid chromosomes have evolved by the pericentric inversions rather than centric fusions. Sometimes, internally located telomeric DNA sequences, unusual distribution of the heterochromatin or redistribution of nuclear organizer regions (NORs) are remnants of such rearrangements (Pisano and Ozuf-Costaz 2000; Sola et al. 2003; Ruiz-Herrera et al. 2008; Rosa et al. 2012). Thus, in the present paper we propose to apply conventional and molecular cytogenetic methods in the European grayling chromosomes in order to find potentially relics of the chromosomal rearrangements accompanying evolution of the *T. thymallus* karyotype.

## Materials and methods

### Materials

Nineteen 1 year old European grayling individuals from the broodstock reared at the Opole fish farm, were sampled for the cytogenetic analysis. The broodstock was established from the spawners caught in Biała Głuchołaska river (South-Western Poland) in 2010. Experimental fish were reared in the recirculating aquaculture system (RAS) in the Aquaculture and Environmental Engineering Centre, University of Warmia and Mazury in Olsztyn. All the manipulations and the experimental procedures were provided according to the positive opinion No 67/2012 of the Local Ethical Commission from the University of Warmia and Mazury in Olsztyn, Poland.

### Chromosome dissection and banding

Fish individuals were injected with 0.1 % colchicine solution (1 mL/100 g body mass, USBiological) 1.5 h before sacrifice. Kidneys were removed and dismembered in 0.075 mol/L KCL. After 45 min of hypotonization cell suspensions were fixed in 3:1 methanol-acetic acid fixative, washed twice in fixative, and spread onto the microscopic slides. Fish were sexed by the microscope analysis of the gonadal tissue.

The C-positive heterochromatin blocks were visualised by C-banding technique as described by Haff and Schmid (1984). Restriction endonuclease *Alu* I (Promega) suspended in the deionized water and appropriate buffer was added to the freshly prepared slides and covered with a 24 × 32 mm coverslips. The amount of the enzyme was 30U per slide. Slides were incubated in a moist chamber at 37 °C for 1–3 h. The optimal incubation time for *Alu* I was 2 h. After incubation, the slides were washed with distilled water, and mounted in the Vectashield antifade reagent containing 4′,6-diamidino-2-phenylindole (DAPI) (1.5 μg/mL) (Vector, Burlingame, USA). Base specific fluorochromes were used in order to characterize composition of the chromosomal DNA. AT-rich heterochromatin regions were identified with DAPI staining by dropping the antifade solution containing DAPI onto a slide and covering it with a coverslip. Chromomycin A_3_ and AgNO_3_ staining were performed for visualization of the GC-rich chromatin and identification of the active NORs, respectively. Chromomycin A_3_ and AgNO_3_ staining were performed as described by Sola et al. (1992) and Howell and Black (1980).

### Fluorescence in situ hybridization (FISH)

Chromosomal localization of the telomeric (TTAGGG)_n_ DNA sequences on the European grayling chromosomes was performed using fluorescence in situ hybridization (FISH) with peptide nucleic acid (PNA)-telomere probe and primed in situ labelling (PRINS) with (CCCTAA)_7_ primer. FISH using fluorescein isothiocyanate (FITC)-conjugated telomere PNA probe (DAKO, Glostrup, Denmark) was carried out according to the manufacturer’s protocol. Slides with metaphase spreads were washed with TBS buffer (Tris-buffered saline, pH 7.5) for 2 min, immersed in 3.7 % formaldehyde in 1 × TBS for 2 min, washed twice in TBS for 5 min each and treated with Pre-Treatment solution including Proteinase K (DAKO) for 10 min. Afterwards, slides were washed twice in TBS buffer for 5 min each, dehydrated through a cold (−20 °C) ethanol series (70, 85, 99.6 %) for 1 min each and air-dried at room temperature. Ten μl of FITC PNA telomere probe mix (DAKO) was dropped on the prepared slides and covered with the coverslip. Chromosomal DNA was denatured at 85^o^ C for 5 min under the coverslip in the presence of the PNA probe. Hybridization took place in the darkness at room temperature for 90–120 min. After hybridization, the coverslips were gently removed in the course of brief immersion in Rinse Solution (DAKO) for 1 min. Slides were washed in Wash Solution (DAKO) for 5 min at 65 °C and dehydrated by immersion through a series of cold ethanol washes of 70, 85, and 99,6 % for 1 min each and air-dried at room temperature. For counterstaining, chromosomes were mounted in the antifade reagent (Vectashield) containing DAPI (Vector).

### Primed in situ labelling (PRINS)

The PRINS reaction was conducted according to Ocalewicz et al. (2004) with some modifications. Slides with metaphase spreads were placed on a 96 °C hotplate for 1 min, then 50 μl of reaction mixture was added, slides were covered with coverslips and left for 3 min on the hotplate. Afterward, slides were transferred to a humid chamber in which they remained for 45 min at 61 °C to anneal the primers and extend the new, labelled DNA sequence. The PRINS reaction mixture consisted of dATP, dGTP, dCTP, and Fluorescein-12-dUTP (Roche, Mannheim, Germany) (0.5 μl each), 2.5 μl of glycerol (Sigma), 3 μl of telomere (CCTAAA)_7_ primer (100 pmol/μl), 5 μl of Taq polymerase buffer, 0.5 μl of Taq polymerase (5U/μl) (Biotools, Madrid, Spain) and 37 μl of dH_2_O. After extension, slides were transferred to stop buffer (50 mM EDTA, 50 mM NaCl, pH = 8) for 5 min at 65 °C, rinsed 3 times in a washing buffer (4XSSC/0,005 % Tween 20) (ICN, Biomedicals, Aurora, USA), pH = 7 for 5 min, and once for 1 min in PBS at room temperature. Chromosomes were counterstained with the antifade solution (Vectashield) containing DAPI (Vector).

### Image processing

Metaphase spreads were examined under a Zeiss Axio Imager A1 microscope equipped with a Zeiss EC Plan-Neofluar 100x/1.3 oil objective, a fluorescent lamp and a digital camera (Applied Spectral Imaging, Galilee, Israel). Images were captured and the electronic processing of the images was performed using the Band View/FISH View software (Applied Spectral Imaging).

## Results

**Fig. 1:**
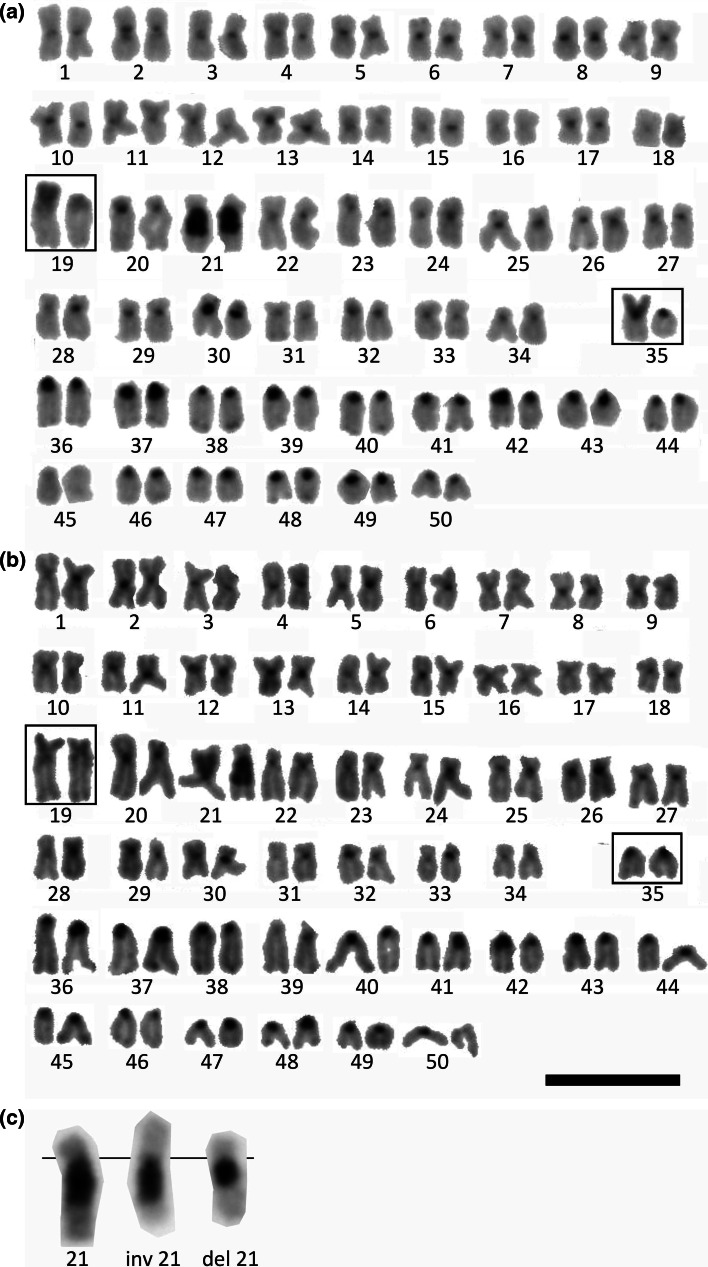
Karyotype of the European grayling stained by C-banding. **a** karyotype: 36 m + 32sm + 32a, genotype of NOR-bearing chromosomes: FfS*s (*framed*); **b** karyotype: 37 m + 31sm + 32a, genotype of NOR-bearing chromosomes: FFss (*framed*), one of homologues of chromosome 21 is inverted form; **c** polymorphism of 21 pair chromosomes: 21—the most common form of the chromosome, del—chromosome with deletion of the heterochromatin, inv—chromosome after inversion.* Scale bar* = 10 μm

**Fig. 2:**
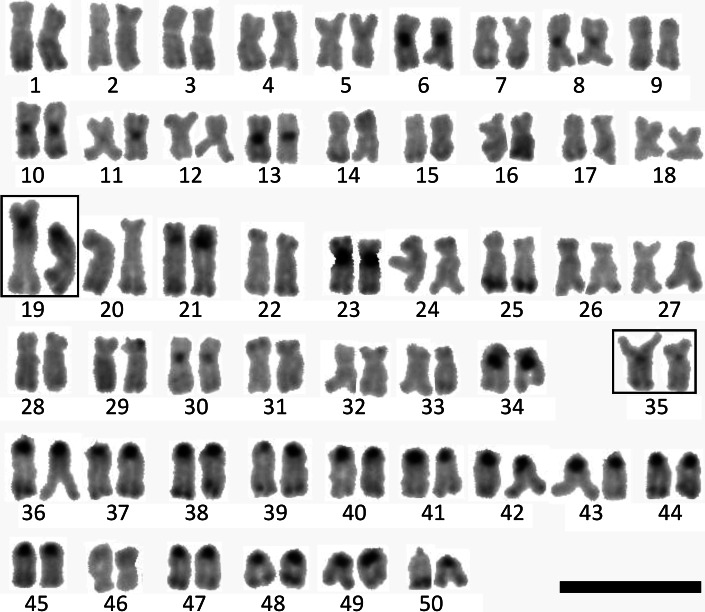
Karyotype of the European grayling. Chromosomes digested by *Alu*I restriction enzyme. The karyotype: 37 m + 32sm + 31a, genotype of NOR-bearing chromosomes: FfS*S (*framed*). *Scale bar* = 10 μm

The karyotype of the European grayling (*T. thymallus*) (2n = 100) comprised 67–70 metacentric (m)-submetacentric (sm) chromosomes and 30–33 subtelocentric (st)-acrocentric (a) chromosomes (NF = 167–170) (Figs. 1, 2). Chromosome arm number variation was due to the length polymorphism of the chromosomes No 19 and 35.

C-banding revealed almost all European grayling chromosomes had heterochromatin blocks at the centric location. Only chromosome No 17 did not show such located C-positive heterochromatin. The largest heterochromatic region was found at the proximal region of the long (q) arm of the chromosome No 21 and showed variation in size and location (Fig. 1a, b). Three cytotypes of this chromosome have been found: (1) sm chromosome with large q-arm heterochromatin, (2) m chromosome with large q-arm heterochromatin and (3) small sm chromosomes with relatively smaller q-arm heterochromatic region (Fig. 1c). The first cytotype was the most frequently observed among the studied individuals (Table 1). The entire short arms of the polymorphic chromosomes No 19 and 35 were also built with the C-banded heterochromatin. Additionally, chromosome No 19 exhibited C-positive heterochromatic block at the proximal region of the q arm. Distinct heterochromatic blocks were observed to cover whole short arms of most of the st-a chromosomes (Fig. 1).

**Table 1:** Polymorphism of three chromosome pairs in the karyotype of *T. thymallus* individuals from Poland (more information in the text)

Fish no.	Sex	Number of Ag/CMA_3_ signals	Genotype of the		
			NOR-bearing pair No 19	NOR-bearing pair No 35	Pair No 21
lipien 01	m	2	FF	ss	inv 21; inv 21
lipien 02	m	3	F*F*	Ss	21; del 21
lipien 03	f	4	FF	S*S	21; del 21
lipien 04	m	2	FF	ss	21; inv 21
lipien 05	f	2	F*F	ss	21; inv 21
lipien 06	m	1	F*f	ss	21; inv 21
lipien 07	f	3	F*F	S*s	21; del 21
lipien 08	m	3	Ff	S*S*	21; inv 21
lipien 10	f	1	ff	S*s	21; del 21
lipien 11	f	2	Ff	Ss	21; inv 21
lipien 12	m	2	Ff	S*s	21; 21
lipien 13	f	3	F*F	Ss	21; 21
lipien 14	m	3	F*F	Ss	21; inv 21
lipien 15	m	2	F*f	S*s	21; inv 21
lipien 16	f	3	F*F	Ss	del 21; inv 21
lipien 17	m	3	Ff	S*S*	21; del 21
lipien 18	m	3	Ff	S*S	21; del 21
lipien 19	m	2	F*f	S*s	21; del 21
lipien 20	f	3	Ff	SS	21; del 21

**Fig. 3:**
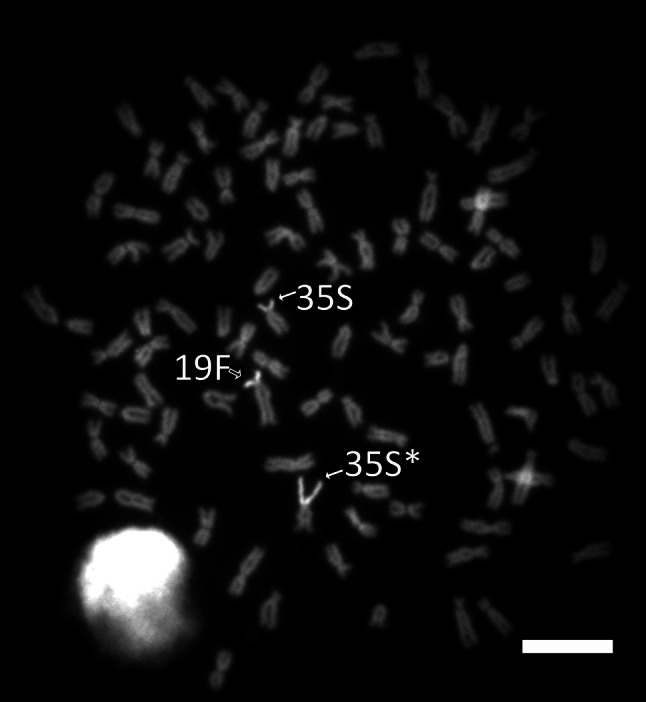
Chromosomes of the European grayling stained with Chromomycin A_3_. *Arrows* indicate NOR-bearing chromosomes 19F (*black arrow*), 35S* and 35S (*white arrows*). *Scale bar* = 10 μm

Centromeric heterochromatin of five metacentric chromosome pairs, five sm chromosome pairs and all st-a chromosomes except chromosome No 46 was resistant to *Alu*I treatment. Moreover, *Alu*I resistant bands were seen at the telomeric regions on the long arms of the chromosome No 25 and six pairs of the a chromosomes (Fig. 2). While the whole heterochromatic short arms of the chromosome pairs No 19 and 35 were digested by *Alu*I endonuclease, C-positive heterochromatic block observed at the proximal region of the long arm of the chromosome 19 remained undigested after *Alu*I treatment (Figs. 2, 3).

The NORs as visualized by Ag NO_3_ and CMA_3_ staining covered almost entire heterochromatic short arms of the chromosomes No 19 and 35 (Fig. 3). Both NOR sites were polymorphic in size as it was described in another grayling stock from Poland by Jankun et al. (2003). Specimens analysed here displayed NOR amplification on one or both homologues. Three forms of each of the NOR bearing chromosomes were observed within studied individuals: (1) chromosomes with double amount of the NOR related heterochromatin (19F* and 35S*), (2) chromosomes with single amount of NORs associated heterochromatin (19F and 35S) and (3) chromosomes with very small or without NOR sites which are subtelocentrics and do not produce Ag/CMA_3_ signals (19f and 35s). Different morphology of the NOR-bearing chromosomes reflected size variation of the NOR related heterochromatin (Fig. 3). The number of silver nitrate/CMA_3_ stained chromosomes varied among individuals from one to four, three being the most prevalent (Table 1). Simultaneous expression of all NOR sites in the same metaphase was rarely observed. NOR sites appeared stable within each individual because staining with Ag/CMA_3_ signals overlapped in all metaphase plates of any individuals.

**Fig. 4:**
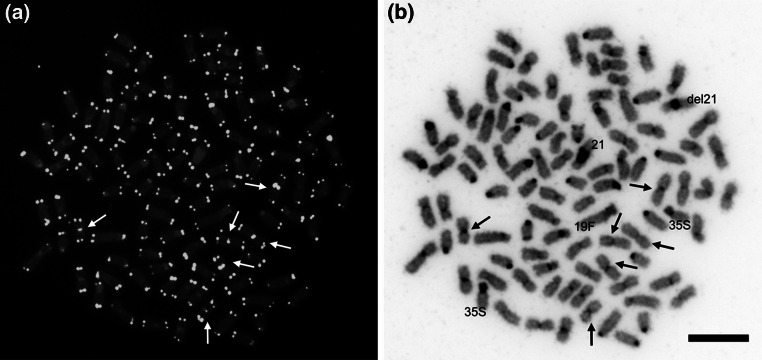
Metaphase spreads of the European grayling after: **a** PNA-FISH with telomeric probe (*bright signals*); **b** DAPI staining. *Arrows* indicate chromosomes with the interstitial telomeric sites. *Scale bar* = 10 μm

Application of FISH with PNA telomere probe as well as PRINS with (CCTAAA)_7_ primer revealed telomeric signals at the ends of the sister chromatids from all chromosomes. Moreover, four medium-sized m chromosomes and two smaller m chromosomes exhibited interstitial telomeric hybridization signals observed at the pericentromeric regions (Fig. 4). In the four medium sized m chromosomes, the interstitial fluorescence spots were easy observed on both of the sister chromatids as two separate signals, while hybridization telomeric signals on the sister chromatids from the two smaller chromosomes were usually observed as single spots. Intensity of the fluorescence interstitial hybridization spots varied between the chromosomes. In two of the medium sized m chromosomes the internal telomeric fluorescence signals were stronger when compared to another chromosomes with the telomeric signals located far from their termini.

Chromosomes denatured at 85 and 96 °C during the FISH and PRINS procedures, respectively, and subsequently stained with DAPI fluorochrome showed banding pattern similar to C-banding pattern. Application of DAPI to counterstain chromosomes after FISH and PRINS exhibited that interstitial telomeric sites were not co-localized with any large blocks of the C-band positive heterochromatin.

## Discussion

C-banding and restriction enzyme digestion applied for the first time in the European grayling chromosomes enabled characteristics of the heterochromatin variation in this species. Structural and length polymorphism of the chromosome 21 showing a conspicuous heterochromatin block adjacent to the centromere seems to be the result of the chromosome rearrangements (Fig. 1). The most frequently observed cytotype (21) could be considered as a plesiomorphic form of the chromosome No 21 (Table 1). Chromosomal morphology, size and location of the heterochromatic segments suggested m and small sm cytotypes of the chromosome 21 appeared in the course of the pericentric inversion (inv 21) and partial deletion of the heterochromatic block (del 21), respectively. Such rearrangements have been described in many groups of organisms as the heterochromatin is consisted of many repetitive DNA sequences and transposable elements that are involved in the chromosome restructuring (Bartolome et al. 2002; Gross et al. 2009; Valente et al. 2011). The repetitive nature of the ribosomal DNA sequences could lead to the length polymorphism of the NOR associated C-banded heterochromatin in the European grayling. As evidenced by the FISH with 18S rDNA probe performed earlier, the European graylings from the Northern Poland has two loci of the major rRNA genes that exactly overlap with the AgNOR and GC-specific fluorochrome (CMA_3_) positively stained segments (Jankun et al. 2003).

To retain chromosome number close to the karyotype of the hypothetical tetraploid ancestor of the salmonids and to increase chromosome arm number, thymallid karyotype experienced numerous pericentric inversions (Phillips and Ráb 2001) and ITSs observed at the pericentromeric regions of the six European grayling m chromosomes are likely relics of the these rearrangements (Fig. 4). Telomeric DNA sequences internally inserted in the course of the pericentric inversions are not so frequently observed in the vertebrates (Pellegrino et al. 1999). In fishes, such mechanism was proposed by Rosa et al. (2012) to explain some of the internal telomeric sites (ITSs) observed in the chromosomes of *Rineloricaria lima* (Siluriformes: Loricaridae). However, internally located telomeric DNA sequences do not necessarily need to be relics of any ancient chromosomal rearrangements. Telomeric or telomeric like DNA sequences are components of the satellite DNA in some vertebrates including fish (Garrido-Ramos et al. 1998). In several fish species, (TTAGGG)_n_ sequences have been found to scatter along the entire fish chromosomes, chromosomal arms or their particular segments like NORs (Ocalewicz 2013; Schneider et al. 2013). TTAGGG sequences may be also inserted into the interstitial positions by the telomerase or transposition of the (TTAGGG)_n_ fragment to repair the DNA double strand breaks (DSBs) (Ruiz-Herrera et al. 2008; Ocalewicz 2013).

Most of the European grayling bi-armed chromosomes displayed telomeric hybridization signals only at the ends of the chromatids. This might suggest that chromosome breaks preceding the pericentric inversions in the graylings were located out of the telomeric regions. On the other hand, if the chromosome breakage occurred within the telomeric region, TTAGGG sequences interstitially inserted via the inversion process may have undergone a gradual loss what could preclude their cytogenetic detection (Slijepcevic 1998).

In fishes like in other vertebrate species, ITSs are detected within or at the margin of the distinct blocks of the C-band positive heterochromatin (Meyne et al. 1990; Ruiz-Herrera et al. 2008; Milhomem et al. 2008; Cioffi et al. 2010), might be observed in the vicinity of the NORs or even to coincide with the NORs (Gornung et al. 2004; Ocalewicz et al. 2004; Pomianowski et al. 2012) or co-localized with 5S ribosomal loci (Rosa et al. 2012). However, this might not be a case here as the European grayling chromosomes exhibiting large heterochromatic segments (chromosome 21) and both NOR bearing chromosomes did not show any internally located telomeric DNA repeats (Fig. 4). Moreover, 5S rDNA loci were found at the pericentromeric region of the six a and only one small m chromosomes in the European grayling (Jankun et al. 2003).

In conclusion, karyotype of the graylings (Thymallinae) is proposed to experienced numerous pericentric inversions and ITSs observed at the pericentromeric regions of the six European grayling metacentric chromosomes are likely relics of the these rearrangements. Although, studied individuals exhibited huge variation in size and location of the C-banded and NOR related heterochromatin none of the ITSs has been observed to coincide either with the large heterochromatin blocks or NOR regions.
